# Prognostic factors for severe *Pneumocystis jiroveci* pneumonia of non-HIV patients in intensive care unit: a bicentric retrospective study

**DOI:** 10.1186/s12879-016-1855-x

**Published:** 2016-09-29

**Authors:** Li Weng, Xu Huang, Lie Chen, Li-Qin Feng, Wei Jiang, Xiao-Yun Hu, Jin-Min Peng, Chun-Yao Wang, Qing-Yuan Zhan, Bin Du

**Affiliations:** 1 Medical ICU, Peking Union Medical College Hospital, Peking Union Medical College and Chinese Academy of Medical Sciences, 1 Shuaifuyuan, Dongcheng district, Beijing, 100730 China; 2 Department of Respiratory and Critical Care Medicine, China-Japan Friendship Hospital, 2 Yinghua Dongjie, Hepingli, Beijing, 100029 China; 3 Department of General Internal Medicine, Shijingshan hospital, Capital Medical University, 24 Shijingshan street, Shijingshan district, Beijing, 100043 China; 4 Intensive Care Unit, Fifth Hospital of Shi Jia Zhuang, 42 Tanan street, Yuhua district, Shijiazhuang, 050021 China

**Keywords:** *Pneumocystis* pneumonia, Intensive care units, Mortality

## Abstract

**Background:**

*Pneumocystis jiroveci* pneumonia (PJP) in non-HIV patients is still a challenge for intensivists. The aim of our study was to evaluate mortality predictors of PJP patients requiring Intensive care unit (ICU) admission.

**Methods:**

Retrospectively review medical records of patients with diagnosis of PJP admitted to four ICUs of two academic medical centers from October 2012 to October 2015.

**Results:**

Eighty-two patients were enrolled in the study. Overall hospital mortality was 75.6 %. Compared with survivors, the non-survivors had older age (55 ± 16 vs. 45 ± 17, *p* = 0.014), higher APACHE II score (20 ± 5 vs. 17 ± 5, *p* = 0.01), lower white blood cell count (7.68 ± 3.44 vs. 10.48 ± 4.62, *p* = 0.005), less fever (80.6%vs. 100 %, *p* = 0.033), more hypotension (58.1 % vs. 20 %, *p* = 0.003), more pneumomediastinum (29 % vs. 5 %, *p* = 0.027). Logistic regression analysis demonstrated that age [odds ratio (OR)1.051; 95 % CI 1.007-1.097; *p* = 0.022], white blood cell count [OR 0.802; 95 % CI 0.670-0.960; *p* = 0.016], and pneumomediastinum [OR 16.514; 95 % CI 1.330-205.027; *p* = 0.029] were independently associated with hospital mortality.

**Conclusions:**

Mortality rate for non-HIV PJP patients requiring ICU admission was still high. Poor prognostic factors included age, white blood cell count and pneumomediastinum.

**Electronic supplementary material:**

The online version of this article (doi:10.1186/s12879-016-1855-x) contains supplementary material, which is available to authorized users.

## Background

With the widespread use of *Pneumocystis jirovecii* pneumonia (PJP) prophylaxis and highly active antiretroviral therapy (HAART), the incidence and mortality of PJP in HIV patients have declined substantially in Europe and the United States [1, 2]. Current estimates of hospital mortality for PJP in HIV patients range from 7 % to 11 % [3]. Most importantly, respiratory failure due to PJP requiring ICU admission was less common in HIV patients [4]. In contrast, PJP rate is increasing in non-HIV patient [5] and the reported mortality of PJP in immunocompromised non-HIV patients ranges from 48 % to 67 % [3]. The clinical course, and inflammatory response might contribute to the different mortality rates between the two groups. In addition, the sample sizes of previous studies exploring the prognostic factor were small [6–8]. The prognostic factor for severe PJP in the intensive care unit (ICU) setting has not been well described.

In order to determine the prognostic factors for PJP in ICU setting, we retrospectively collected data for a consecutive series of PJP patients requiring ICU admission from October 2012 to October 2015.

## Methods

### Study design

We conducted a bicentric retrospective cohort study in 4 ICUs at 2 academic medical centers, including a medical ICU, an emergency ICU, and a general ICU at Peking Union Medical College Hospital (PUMCH 1800 beds), and a medical ICU at China-Japan Friendship Hospital (CJFH 1610 beds). All four ICUs contain 83 beds during the study period.

All patients discharged with diagnosis of PJP from October 2012 to October 2015 were screened for eligibility. Inclusion criteria: (1) PJP, confirmed by Polymerase Chain Reaction (PCR) or methenamine silver stain of samples from bronchoalveolar lavage fluid (BALF), aspirate or sputum; (2) HIV tests negative; (3) ICU admission during index hospitalization due to respiratory insufficiency.

Patients without immunocompromised background, less than 18 years old or pregnant were excluded.

### Data collection

Data were gathered retrospectively from medical records at both PUMCH and CJFH by the investigators (L.C., L-Q.F., L.W., and X.H.). Patient identifiers were removed from the final data sheet and were coded with a numbered assignment.

Demographic, laboratory, and clinical data were collected, including age, gender, microbiological findings for PJP, severity of illness based on the acute physiology and chronic health evaluation (APACHE) II score, comorbidities, complications, the time of PJP symptom onset, the time of PJP diagnosis, the time to appropriate antibiotics administration, and the chest radiographs and CT scan findings. Data on mechanical ventilation included type of oxygen therapy or ventilatory support on ICU admission; tidal volume, plateau pressure, positive end-expiratory pressure (PEEP), fraction of inspired oxygen (FiO2) and recruitment maneuver on ICU admission; maximal tidal volume, maximal PEEP during ICU stay. Hospital mortality was the primary outcome of our study.

### Definition

(1) PJP was defined as: symptoms and radiographs compatible with PJP [9]; confirmed by PCR or methenamine silver stain of samples from bronchoalveolar lavage fluid (BALF), aspirate or sputum. (2) ventilator-associated pneumonia (VAP) was defined as a new lung parenchymal opacity on a chest radiograph of a patient intubated for more than 48 h; and simultaneous presentation of two or more of: purulent secretion from tracheal; temperature of less than 36 °C or more than 38 °C; white blood cell count (WBC) of less than 4 × 109/L or more than 10 × 109/L. (3) Pulmonary aspergillosis was defined by one host factor criterion, one microbiological criterion and one major clinical criterion (or 2 minor criteria) according to an international consensus from experts of the European Organization for Research and Treatment of Cancer (EORTC) and the National Institute of Allergy and Infectious Diseases Mycoses Study Group [10]. (4) Cytomegalovirus (CMV) infection was confirmed if pp65-antigenemia assay or CMV DNA assay were positive [11]. (5) Barotrauma was defined as pneumomediastinum or pneumothorax. (6) Pneumomediastinum was diagnosed as the presence of free air in the mediastinal cavity by CT scan or Chest x-ray showing hyperlucent lines outlining the lateral heart borders with subcutaneous emphysema around neck and chest region.

This study was approved by the institutional review board of Peking Union Medical College Hospital (S-K116). Due to the retrospective nature of the study, informed written consent was waived.

### Statistical analysis

For the statistical analysis, continuous data were compared with use of the Student’s *t* test or Mann-Whitney test as appropriate. Statistical analysis of non-continuous dichotomous data was compared by the chi-square test or the Fisher’s Exact Test as appropriate. Logistic regression models were used to determine the effect of prognostic factors on hospital death by means of stepwise backward elimination procedures, after adjusting for covariates of which the p values were less than 0.1. All statistical analyses were performed using SPSS statistical software (version 22.0; IBM Inc., Armonk, NY). All p values were two sided, and statistical significances were accepted for p < 0.05.

## Results

**Fig. 1:**
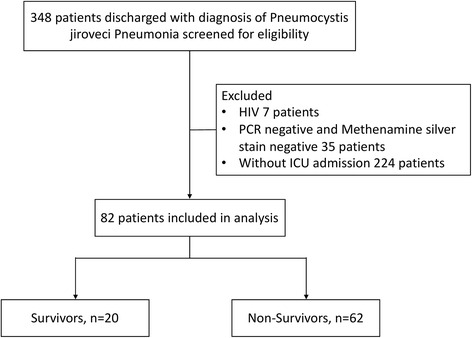
The patient flowchart with respect to inclusion and exclusion

Overall, 348 patients were discharged with a diagnosis of PJP during the study period, of whom 266 met exclusion criteria. Consequently, 82 patients were available for the final analysis, including 72 patients PUMCH and 10 patients in CJFH (Fig. 1).

Table 1 shows characteristics and outcomes of confirmed PJP in non-HIV patients. The entire 82 patient cohort had a mean age 53 ± 17 years of and APACHE II score of19 ± 5, with 41.4 % male. Most of the patients had an underlying disease of connective tissue disease (79.3 %) and history of corticosteroid therapy (84.1 %). All patients were treated with trimethoprim-sulfamethoxazole (TMP-SMX). Not a single patient received PJP prophylaxis. PJP was diagnosed by methenamine silver stain, PCR, or both in 13, 45, and 24 patients respectively, without any difference between 2 hospitals (Additional file 1: Table S2). PJP-PCR positive samples included sputum (*n* = 5), trachea aspirate (*n* = 17), and BALF (*n* = 49). Methenamine silver stain positive samples included sputum (*n* = 4), trachea aspirate (*n* = 12), and BALF (*n* = 19). All pulmonary aspergillosis was diagnosed as EORTC probable invasive aspergillosis with the presence of a host factor, a clinical criterion, and a mycological criterion. For the 38 patients receiving caspofungin, total days on caspofungin were 8 ± 6. Twenty-five patients received empirical caspofungin therapy for less than 7 days for suspected invasive fungal infection. Another 6 patients received caspofungin as a combination therapy with amphotericin B or voriconazole for aspergillosis. Although the clinical efficacy of caspofungin as salvage therapy for PJP remained controversial [12], it was administered in 7 patients in our cohort study as a salvage regimen of whom 5 patients died during study period.

**Table 1:** Characteristics and outcomes of confirmed pneumocystis jiroveci pneumonia in non-HIV patients

	Survivors	Non-survivors	*P* value
	*N* = 20	*N* = 62	
Age, mean ± SD	45 ± 17	55 ± 16	0.014
Male, n (%)	6(30.0)	28(45.2)	0.231
Apache II, mean ± SD	17 ± 5	20 ± 5	0.010
Underlying disease			
CTD, n (%)	16(80.0)	49(79.0)	0.926
ILD, n (%)	1(5.0)	1(1.6)	0.431
Dermatomyositis, n (%)	1(5.0)	9(14.5)	0.438
Organ transplant, n (%)	2(10.0)	1(1.6)	0.146
Hematologic malignancy, n (%)	0(0)	3(4.8)	0.427
Solid tumor, n (%)	2(10.0)	2(3.2)	0.249
Corticosteroid therapy, n (%)	17(94.4)	52(89.7)	0.539
Symptom			
Fever, n (%)	20(100)	50(80.6)	0.033
Dyspnea, n (%)	19(95.0)	56(90.3)	0.515
Cough, n (%)	15(75)	42(67.7)	0.540
Microbiological methods			
PCR, n (%)	17(85.0)	54(87.1)	0.811
Methenamine silver stain, n (%)	11(55.0)	24(38.7)	0.200
Repiratory samples			
Sputum, n (%)	0(0)	6(9.7)	0.148
Trachea aspirate, n (%)	6(30.0)	16(25.8)	0.713
Bronchoalveolar lavage, n (%)	14(70.0)	40(64.5)	0.653
Laboratory findings			
White blood cell counts, mean ± SD	10.48 ± 4.62	7.68 ± 3.44	0.005
lymphocyte counts, mean ± SD	678 ± 600	514 ± 441	0.117
CD4 cell counts, mean ± SD	215 ± 225	159 ± 343	0.510
PaO2/FiO2 on ICU admission	161 ± 69	131 ± 65	0.064
Radiographic findings			
Ground glass opacities, n (%)	20(100.0)	62(100.0)	
Bilateral symmetric, n (%)	17(85.0)	55(88.7)	0.700
Co-infections			
Bacteremia, n (%)	2(10.0)	6(9.7)	0.966
VAP, n (%)	5(25.0)	27(43.5)	0.139
Aspergillosis, pulmonary, n (%)	5(25.0)	13(21.0)	0.705
Cytomegalovirus, n (%)	10(50.0)	40(64.5)	0.247
Complications			
Hypotension, n (%)	4(20.0)	36(58.1)	0.003
NE(mcg/kg/min), mean ± SD	0.22 ± 0.16	0.39 ± 0.36	0.439
Barotrauma			
Pneumothorax, n (%)	2(10.0)	9(14.5)	0.465
Pneumomediastinum, n (%)	1(5.0)	18(29)	0.027
Intervals			
Onset to diagnosis, days, mean ± SD	15 ± 12	14 ± 10	0.488
Onset to intubation, days, mean ± SD	10 ± 6	12 ± 11	0.786
Onset to TMP/SMZ, days, mean ± SD	14 ± 18	11 ± 10	0.538
Respiratory support			
IPPV during ICU stay, n (%)	16(80.0)	59(95.2)	0.057
NPPV on ICU admission, n (%)	4(20.0)	8(12.9)	0.474
IPPV on ICU admission, n (%)	10(50.0)	36(58.1)	0.527
NRM on ICU admission, n (%)	6(30.0)	18(29.0)	0.934
Medication			
Adjunctive steroid, n (%)	16(80.0)	48(77.4)	0.808
Caspofungin, n (%)	7(35.0)	31(50.0)	0.242

Values are expressed as the mean ± SD or Number (%), unless otherwise indicated. *CTD* Connective Tissue Disease, *ILD* Interstitial lung Disease, *PCR* polymerase chain reaction, *BALF* bronchoalveolar lavage fluid, *VAP* ventilator-associated pneumonia, *NE* norepinephrine, *TMP/SMZ* trimethoprim-sulfamethoxazole, *IPPV* Invasive positive pressure ventilation, *NPPV* noninvasive positive pressure ventilation, *NRM* non-rebreathing mask

During their hospital stay, 62 (75.6 %) of the 82 patients died. Compared with survivors, the non-survivors had older age (55 ± 16 vs. 45 ± 17, *p* = 0.014), higher APACHE II score (20 ± 5 vs. 17 ± 5, *p* = 0.01), lower WBC (7.68 ± 3.44 vs. 10.48 ± 4.62, *p* = 0.005), less fever (80.6%vs. 100 %, *p* = 0.033), more hypotension (58.1 % vs. 20 %, *p* = 0.003), and more pneumomediastinum (29 % vs. 5 %, *p* = 0.027), while the difference was not statistically significant for lymphocyte counts, CD4 cell count, type of respiratory support on ICU admission. Four patients received high-frequency oscillatory ventilation and one patient received extracorporeal membrane oxygenation. All these five patients died during the hospital stay.

To investigate the role of potential confounding prognostic factors, a multivariate analysis was performed for hospital mortality (Table 2). The Hosmer and Lemeshow goodness-of-fit test were not rejected (*p* = 0.640), indicating adequate model fit. No interaction terms were found to be significant in this model, and there was no collinearity between any of the independent variables. The multivariate model indicated that age [odds ratio (OR)1.051; 95 % CI 1.007-1.097; *p* = 0.022], WBC [OR 0.802; 95 % CI 0.670-0.960; *p* = 0.016], and pneumomediastinum [OR 16.514; 95 % CI 1.330-205.027; *p* = 0.029] were independently significantly associated with hospital mortality.

**Table 2:** Multivariate analysis for predictors of death in patients with confirmed pneumocystis jiroveci pneumonia in non-HIV patients

	Multivariate analysis	Wald stat.	*P* value
	Odds Ratio (95 % CI)		
Age	1.051(1.007-1.097)	5.238	0.022
White blood cell counts	0.802 (0.670-0.960)	5.787	0.016
Pneumomediastinum	16.514(1.330-205.027)	4.761	0.029

The risk factors removed from the logistic regression model including: APACHE II; Fever; PaO2/FiO2 on ICU admission; IPPV on ICU admission; Hypotension

CT scans were performed for all those 82 patients on ICU admission. Pneumomediastinum was confirmed by CT in 14 patients and chest x-ray plus subcutaneous emphysema in 5 patients. Six patients developed pneumomediastinum before hospital admission. Other 13 patients had time intervals between ICU admission and pneumomediastinum with a mean of 9 days (range 2–30 days). The potential risk factors contributed to pneumomediastinum were listed in Table 3. There was statistically significant difference in the percentage of patients treated with non-rebreathing mask (NRM) on ICU admission between pneumomediastinum and non-pneumomediastinum (47.4 % vs. 23.8 %, *p* = 0.048). Tidal volume, plateau pressure, and PEEP was similar between those two groups.

**Table 3:** Risk factors for Pneumomediastinum of confirmed pneumocystis jiroveci pneumonia in non-HIV patients

	Pneumomediastinum	Non-Peumomediastinum	*P* value
	*N* = 19	*N* = 63	
Age, mean ± SD	51 ± 19	53 ± 16	0.606
Male, n (%)	8(42.1)	26(41.3)	0.948
Apache II, mean ± SD	19 ± 5	19 ± 5	0.948
Underlying conditions			
CTD, n (%)	16(84.2)	49(77.8)	0.544
ILD, n (%)	1(5.3)	1(1.6)	0.412
Dermatomyositis, n (%)	1(5.3)	9(14.3)	0.440
Organ transplant, n (%)	1(5.3)	2(3.2)	0.552
Hematologic malignancy, n (%)	0(0)	3(4.8)	0.448
Solid tumor, n (%)	1(5.3)	3(4.8)	0.659
Corticosteroid therapy, n (%)	17(89.5)	52(82.5)	0.722
Symptom			
Fever, n (%)	17(89.5)	53(84.1)	0.563
Dyspnea, n (%)	19(100)	56(88.9)	0.192
Cough, n (%)	14(73.7)	43(68.3)	0.652
Laboratory findings			
White blood cell counts, mean ± SD	7.85 ± 2.92	8.52 ± 4.18	0.518
lymphocyte counts, mean ± SD	601 ± 618	540 ± 444	0.717
CD4 cell counts, mean ± SD	246 ± 583	156 ± 186	0.886
PaO2/FiO2 on ICU admission	144 ± 53	136 ± 70	0.663
Co-infections			
Aspergillosis, pulmonary, n (%)	3(15.8)	15(23.8)	0.544
Cytomegalovirus, pulmonary, n (%)	12(63.2)	38(60.3)	0.824
Intervals			
Onset to diagnosis, days, mean ± SD	13 ± 5	14 ± 12	0.480
Onset to intubation, days, mean ± SD	11 ± 6	12 ± 11	0.753
Onset to TMP/SMZ, days, mean ± SD	9 ± 6	12 ± 13	0.230
Respiratory support			
IPPV during ICU stay, n (%)	18(94.7)	57(90.5)	0.560
VT maximal(ml/kg), mean ± SD	7.1 ± 2.0	8.1 ± 2.4	0.208
PEEP maximal(cmH2O), mean ± SD	11 ± 5	10 ± 4	0.415
NPPV on ICU admission, n (%)	3(15.8)	9(14.3)	0.871
IPAP(cmH2O), mean ± SD	14 ± 3	11 ± 3	0.118
EPAP(cmH2O), mean ± SD	7 ± 2	7 ± 2	0.980
IPPV on ICU admission, n (%)	7(36.8)	39(61.9)	0.054
VT(ml/kg), mean ± SD	6.8 ± 1.0	7.1 ± 1.2	0.462
Pplat(cmH2O), mean ± SD	25 ± 4	24 ± 6	0.821
PEEP(cmH2O), mean ± SD	10 ± 4	10 ± 4	1.000
FiO2, mean ± SD	0.74 ± 0.15	0.65 ± 0.18	0.066
Recruitment maneuvers, n (%)	6(35.3)	17(30.9)	0.735
NRM on ICU admission, n (%)	9(47.4)	15(23.8)	0.048

Values are expressed as the mean ± SD or Number (%), unless otherwise indicated. *VT* tidal volume of predicted body weight, *Pplat* plateau pressure, *PEEP* positive end-expiratory pressure, *FiO2* fraction of inspired oxygen, *IPAP* inspiratory positive airway pressure, *EPAP* expiratory positive airway pressure

## Discussion

In this bicentric retrospective observational study across 4 Chinese ICUs, we found that age, WBC, and pneumomediastinum were significantly associated with hospital mortality in non-HIV immunocompromised patients with severe PJP who had been admitted to ICU. Use of non-rebreathing mask might contribute to the development of pneumomediastinum.

The most interesting finding of our study was that pneumomediastinum was associated with increased hospital mortality. Pneumomediastinum is the presence of extra-alveolar air in the mediastinum, which is believed to arise from free air leaking from ruptured alveoli. It was described as an uncommon complication of opportunistic infections in HIV-infected patients [13, 14]. However, 24.4 % patients in our study developed pneumomediastinum. The incidence rate discrepancies may be due to different underlying disease (HIV vs. Non-HIV) and few reported incidence rate in previous studies. A retrospective radiographic analysis reported an incidence rate of 11.1 % (4 of 36) in a cohort of moderate non-HIV PJP patients with a mortality rate of 33.3 % [15]. We also found more pneumomediastinum developed in the NRM group. This might be explained by higher trans-pulmonary pressure and tidal volume during spontaneous breath resulting in air leak, which was consistent with a previous report of HIV patients [13]. Delayed intubation was considered as a risk factor for worse outcome [6]. Although there was no difference in the time interval from symptom onset to intubation in our study, use of non-rebreathing mask instead of positive pressure support on ICU admission suggested delay intubation which was very difficult to define.

Development of pneumothorax was independently associated with increased mortality in previous studies [6, 16]. We did not find any difference in mortality between patients with and without pneumothorax. Protective lung ventilation strategies might account for the different findings. The tidal volumes in our study were smaller than Festic and colleagues (7 ml/kg vs. 10 ml/kg). Despite the application of protective lung ventilation, one fourth patient in this cohort developed pneumomediastinum, which suggested that pneumomediastinum was not a complication of intervention. As Cho et al. [17] reported, the development of the pulmonary cysts and bronchiectasis that were noted in follow up CT but were not visible on CT at admission could be risk factors for development of pneumomediastinum.

We also found lower WBC was related to increased mortality. Although WBC has never been reported as risk factors, previous study [18] suggested a trend of higher WBC in non-HIV patients and survivors, which was consistent with our findings.

Overall mortality of the patients in our study was 75.6 %. Although the reported mortality rates of ICU non-HIV patients with PJP in previous studies were 38.9-84.2 % [6–8, 18–28] (Additional file 2: Table S1), most of the mortality rates were less than 70 %, which were lower than that of our study. The high hospital mortality rate in our study possibly was related to different underlying diseases and no prophylaxis of PJP for those patients. However, although prophylaxis for PJP was recommended for HIV patients, the efficacy of prophylaxis for immunocompromised non–HIV patients has not been well established [29–31], especially for the patients with underlying disease of connective tissue disease. In a recent study of ICU patients with PJP, adjunctive steroid was associated with increased mortality [16]. This might be the cause of high hospital mortality in our study. Considering that most of the patients in our study received steroid therapy before ICU admission, the use of steroid was not avoidable. Moreover, there was no difference in steroid therapy between survivors and non-survivors, and the effects of other covariates remained significant.

In a retrospective study, Chen and colleagues reported the characteristics and prognostic factors of 69 HIV-negative patients with PJP from PUCMH during 10-year study period [25]. In comparison, we had enrolled 72 patients with confirmed PJP during a 3-year period in our cohort (Additional file 1: Table S2, Additional file 3: Table S3). Increasing awareness of the disease in immunocompromised patients among clinicians and widespread implementation of PCR technique for PJP diagnosis might account for the discrepancy between Chen’s study and ours.

### Limitation

The main limitation of the current study was the retrospective nature of the investigation. Considering the relatively low incidence of PJP in ICU, it would be reasonable to prospectively collect data in the future investigation based on current finding. The second limitation was the small population recruited. To our best knowledge, only one study [8] included more ICU PJP patients than ours (88 vs. 82). However, because we focused on the prognostic factors for PJP in an ICU setting, our findings were more helpful to the ICU patients. Third, PJP was diagnosed on the basis of PJP PCR result in some patients. Due to the colonization of PJP, there is a possibility of false positive results. According to recent studies [32, 33], quantitative real-time Polymerase Chain Reaction (PCR) might be helpful in discriminating colonization from infection. However, those real-time PCR was not available in our centers during the study period. Nevertheless, all patients included in analysis had symptoms and their radiographic findings were compatible with PJP.

## Conclusion

Our finding suggested that PJP in non-HIV patients requiring ICU admission remains a challenge for clinician. Poor prognostic factors included older age, lower WBC, and development of pneumomediastinum.

## Additional files

Additional file 1: Table S2. PJP diagnosis by methenamine and PCR in participating centers. (DOCX 59 kb) Additional file 2: Table S1. Summary of published studies. (DOCX 88 kb) Additional file 3: Table S3. Comparison of patients screened for PJP and patient enrollment in 3 studies in Peking Union Medical College Hospital. (DOCX 73 kb)

## Abbreviations

APACHE: Acute physiology and chronic health evaluation
BALF: Bronchoalveolar lavage fluid
CJFH: China-Japan Friendship Hospital
CMV: Cytomegalovirus
EORTC: European Organization for Research and Treatment of Cancer
FiO2: Fraction of inspired oxygen
HAART: Highly active antiretroviral therapy
ICU: Intensive care unit
PEEP: Positive end-expiratory pressure
PJP: Pneumocystis jirovecii pneumonia
PUMCH: Peking Union Medical College Hospital
TMP-SMX: Trimethoprim-sulfamethoxazole
VAP: Ventilator-associated pneumonia
WBC: White blood cell count
