# Supplementary material for: Prognostic factors for severe Pneumocystis jiroveci pneumonia of non-HIV patients in intensive care unit: a bicentric retrospective study
Source: BMC Infect Dis. 2016 Sep 29;16:528. doi: 10.1186/s12879-016-1855-x (PMC5041573; doi:10.1186/s12879-016-1855-x)
Supplement: Additional file 1: Table S2. — PJP diagnosis by methenamine and PCR in participating centers. (DOCX 59 kb) [file 12879_2016_1855_MOESM1_ESM.docx]

e-Table 2. PJP diagnosis by methenamine and PCR in participating centers

| Center | Number of Patients Enrolled | Diagnosis of PJP | | |
| --- | --- | --- | --- | --- |
|  |  | Methenamine silver stain only | PCR only | Both |
| PUMCH | 72 | 13 | 41 | 18 |
| Medical ICU | 51 | 5 | 34 | 12 |
| Surgical ICU | 15 | 5 | 6 | 4 |
| Emergency ICU | 6 | 3 | 1 | 2 |
| CJFH | 10 | 0 | 4 | 6 |
| Total | 82 | 13 | 45 | 24 |

CJFH, China-Japan Friendship Hospital; ICU, intensive care unit; PCR, polymerase chain reaction; PJP, Pneumocystitis jiroveci pneumonia; PUMCH, Peking Union Medical College Hospital.
