# Supplementary material for: Prognostic factors for severe Pneumocystis jiroveci pneumonia of non-HIV patients in intensive care unit: a bicentric retrospective study
Source: BMC Infect Dis. 2016 Sep 29;16:528. doi: 10.1186/s12879-016-1855-x (PMC5041573; doi:10.1186/s12879-016-1855-x)
Supplement: Additional file 2: Table S1. — Summary of published studies. (DOCX 88 kb) [file 12879_2016_1855_MOESM2_ESM.docx]

| e-Table 1, Summary of published studies | | | | | | | |
| --- | --- | --- | --- | --- | --- | --- | --- |
| author | All patients included | Non-HIV patients | ICU Non-HIV patients | ICU Non-HIV patient mortality | All-cause mortality | Statistical analysis | Prognostic factors |
| Mansharamani et al [1], 2000 | 605 | 33 | 23 | 59% | 39% | Univariate | respiratory failure |
| Roblot et al [2], 2002 | 103 | 103 | 58 | 67.2% | 38% | Univariate | high respiratory rate, high pulse rate, elevated C-reactive protein, elevated serum lactate dehydrogenase level, and mechanical ventilation |
| Zahar et al [3], 2002 | 39 | 39 | 39 | 33.3% | 33.3% | Univariate | Receipt of 11 course of chemotherapy, Organ System Failure, SAPS, mechanical ventilation, vasopressor |
| Festic et al [4], 2005 | 30 | 30 | 30 | 67% | 67% | multivariate | intubation delay |
| Monnet et al [5], 2008 | 73 | 27 | 27 | 62% | 41% | multivariate | HIV-negative status, SAPS II |
| Boonsarngsuk et al [6], 2009 | 44 | 30 | 30 | 66.7% | 63.6% | multivariate | corticosteroid use prior to diagnosis of PCP, level of PEEP on day 3 of ARF |
| Hardak et al [7], 2012 | 58 | 58 | 17 | 59% | 17.2% | Univariate | co-infections, high lactate dehydrogenase levels, female gender, and higher pneumonia severity index and acute physiology and chronic health evaluation III scores |
| Roembke et al [8], 2013 | 51 | 30 | 19 | 84.2% | 25.5% | multivariate | ICU, HIV statuses |
| Fillatre et al[9], 2013 | 70 | 70 | 70 | 52.9% | 52.9% | multivariate | ARDS, Herpes virus in bronchoalveolar lavage, Shock, Hemodialysis |
| Guo et al [10], 2014 | 151 | 46 | 18 | 38.9% | 13.2% | multivariate | four or more of the following clinical manifestations (cough, dyspnea, fever, chest pain, and weight loss), ICU |
| Kim et al [11], 2014 | 173 | 173 | 88 | 69.3% | 35.8% | multivariate | High D(Aea)O2, combined bacteremia, increased BUN and preexisting lung disease |
| Li et al [12], 2014 | 43 | 20 | 18 | 66.7% | 32.6% | Univariate | low lymphocyte count, shock |
| Roux et al [13], 2014 | 544 | 321 | 134 | 58.2% | 15.3% | multivariate | Non-AIDS, non-solid organ transplant, age, HSCT, need for oxygen on admission, invasive mechanical ventilation, longer time from admission to initiation of PCP treatment |
| Chen et al [14], 2015 | 69 | 69 | 20 | 70% | 31.9 | multivariate | PaO2/FiO2 ratios, albumin |

1. Mansharamani NG, Garland R, Delaney D, Koziel H: **Management and outcome patterns for adult Pneumocystis carinii pneumonia, 1985 to 1995: comparison of HIV-associated cases to other immunocompromised states.** *Chest* 2000, **118**(3):704-711.

2. Roblot F, Godet C, Le Moal G, Garo B, Faouzi Souala M, Dary M, De Gentile L, Gandji JA, Guimard Y, Lacroix C *et al*: **Analysis of underlying diseases and prognosis factors associated with Pneumocystis carinii pneumonia in immunocompromised HIV-negative patients.** *European Journal of Clinical Microbiology and Infectious Diseases* 2002, **21**(7):523-531.

3. Zahar JR, Robin M, Azoulay E, Fieux F, Nitenberg G, Schlemmer B: **Pneumocystis carinii pneumonia in critically ill patients with malignancy: a descriptive study**. *Clin Infect Dis* 2002, **35**(8):929-934.

4. Festic E, Gajic O, Limper AH, Aksamit TR: **Acute respiratory failure due to pneumocystis pneumonia in patients without human immunodeficiency virus infection: outcome and associated features.** *Chest* 2005, **128**(2):573-579.

5. Monnet X, Vidal-Petiot E, Osman D, Hamzaoui O, Durrbach A, Goujard C, Miceli C, Bourée P, Richard C: **Critical care management and outcome of severe Pneumocystis pneumonia in patients with and without HIV infection.** *Critical care (London, England)* 2008, **12**(1):R28.

6. Boonsarngsuk V, Sirilak S, Kiatboonsri S: **Acute respiratory failure due to Pneumocystis pneumonia: outcome and prognostic factors**. *International Journal of Infectious Diseases* 2009, **13**(1):59-66.

7. Hardak E, Neuberger A, Yigla M, Berger G, Finkelstein R, Sprecher H, Oren I: **Outcome of Pneumocystis jirovecii pneumonia diagnosed by polymerase chain reaction in patients without human immunodeficiency virus infection**. *Respirology* 2012, **17**(4):681-686.

8. Roembke F, Heinzow HS, Gosseling T, Heinecke A, Domagk D, Domschke W, Meister T: **Clinical outcome and predictors of survival in patients with pneumocystis jirovecii pneumonia - results of a tertiary referral centre**. *The Clinical Respiratory Journal* 2013, **8**(1):86-92.

9. Fillatre P, Chevrier S, Revest M, Gacouin A, Jouneau S, Leroy H, Robert-Gangneux F, Minjolle S, Le Tulzo Y, Tattevin P: **Human herpes virus co-infection is associated with mortality in HIV-negative patients with Pneumocystis jirovecii pneumonia**. *Eur J Clin Microbiol Infect Dis* 2013, **32**(2):189-194.

10. Guo F, Chen Y, Yang S-L, Xia H, Li X-W, Tong Z-H: **Pneumocystis Pneumonia in HIV-Infected and Immunocompromised Non-HIV Infected Patients: A Retrospective Study of Two Centers in China**. *PloS one* 2014, **9**(7):e101943.

11. Kim SJ, Lee J, Cho Y-J, Park YS, Lee C-H, Il Yoon H, Lee S-M, Yim J-J, Lee JH, Yoo C-G *et al*: **Prognostic factors of Pneumocystis jirovecii pneumonia in patients without HIV infection**. *Journal of Infection* 2014, **69**(1):88-95.

12. Li M-C, Lee N-Y, Lee C-C, Lee H-C, Chang C-M, Ko W-C: **Pneumocystis jiroveci pneumonia in immunocompromised patients: Delayed diagnosis and poor outcomes in non-HIV- infected individuals**. *Journal of microbiology, immunology, and infection* 2014, **47**(1):42-47.

13. Roux A, Canet E, Valade S, Gangneux-Robert F, Hamane S, Lafabrie A, Maubon D, Debourgogne A, Le Gal S, Dalle F *et al*: **Pneumocystis jirovecii pneumonia in patients with or without AIDS, France**. *Emerg Infect Dis* 2014, **20**(9):1490-1497.

14. Chen M, Tian X, Qin F, Zhou J, Liu J, Wang M, Xu K-F: **Pneumocystis Pneumonia in Patients with Autoimmune Diseases: A Retrospective Study Focused on Clinical Characteristics and Prognostic Factors Related to Death**. *PloS one* 2015, **10**(9):e0139144-e0139144.
