# Supplementary material for: Prognostic factors for severe Pneumocystis jiroveci pneumonia of non-HIV patients in intensive care unit: a bicentric retrospective study
Source: BMC Infect Dis. 2016 Sep 29;16:528. doi: 10.1186/s12879-016-1855-x (PMC5041573; doi:10.1186/s12879-016-1855-x)
Supplement: Additional file 3: Table S3. — Comparison of patients screened for PJP and patient enrollment in 3 studies in Peking Union Medical College Hospital. (DOCX 73 kb) [file 12879_2016_1855_MOESM3_ESM.docx]

e-Table 3: Comparison of patients screened for PJP and patient enrollment in 3 studies in Peking Union Medical College Hospital

|  | Patients screened for PJP | Number of patients enrolled | Diagnosis of PJP | | |
| --- | --- | --- | --- | --- | --- |
| Study period |  |  | Methenamine silver stain only | PCR only | Both |
| Li et al [1] |  |  |  |  |  |
| Jan 2004-May 2005 | N/A | 7 |  |  |  |
|  |  |  |  |  |  |
| Chen et al [2] |  |  |  |  |  |
| Jan 2004-Dec 2004 | 12 | 6 | 1 | 5 | 0 |
| Jan 2005-Dec 2005 | 9 | 5 | 3 | 1 | 1 |
| Jan 2006-Dec 2006 | 15 | 5 | 2 | 0 | 3 |
| Jan 2007-Dec 2007 | 17 | 5 | 0 | 1 | 4 |
| Jan 2008-Dec 2008 | 30 | 11 | 5 | 0 | 6 |
| Jan 2009-Dec 2009 | 23 | 2 | 0 | 0 | 2 |
| Jan 2010-Dec 2010 | 29 | 8 | 3 | 1 | 4 |
| Jan 2011-Dec 2011 | 25 | 6 | 2 | 1 | 3 |
| Jan 2012-Dec 2012 | 43 | 9 | 4 | 3 | 2 |
| Jan 2013-Dec 2013 | 59 | 12 | 4 | 4 | 4 |
|  |  |  |  |  |  |
| Current study |  |  |  |  |  |
| Oct 2012-Dec 2012 | 13 | 5 | 2 | 2 | 1 |
| Jan 2013-Dec 2013 | 59 | 12 | 4 | 4 | 4 |
| Jan 2014-Dec 2014 | 139 | 35 | 1 | 28 | 6 |
| Jan 2015-Oct 2015 | 85 | 20 | 4 | 9 | 7 |

Reference:

1. Li J, Huang X-M, Fang W-G, Zeng X-J: **Pneumocystis carinii pneumonia in patients with connective tissue disease**. *Journal of clinical rheumatology : practical reports on rheumatic & musculoskeletal diseases* 2006, **12**(3):114-117.

2. Chen M, Tian X, Qin F, Zhou J, Liu J, Wang M, Xu K-F: **Pneumocystis Pneumonia in Patients with Autoimmune Diseases: A Retrospective Study Focused on Clinical Characteristics and Prognostic Factors Related to Death**. *PloS one* 2015, **10**(9):e0139144-e0139144.
